# Evaluation of changes in the cognitive function of adult cynomolgus monkeys under stress induced by audio-visual stimulation by applying modified finger maze test

**DOI:** 10.3389/fnins.2022.959174

**Published:** 2022-10-31

**Authors:** Ying Huang, Hong Wang, Chen Yang, Yuchong Luo, Yongyan Ding, Hongjun Jin, Shenglin Wen

**Affiliations:** ^1^Department of Psychology, The Fifth Affiliated Hospital, Sun Yat-sen University, Zhuhai, China; ^2^Hong Kong and Macao Central Nervous Regeneration Research Institute, Ji'nan University, Guangzhou, China; ^3^Guangdong Provincial Key Laboratory of Biomedical Imaging, Guangdong Provincial Engineering Research Center of Molecular Imaging, The Fifth Affiliated Hospital, Sun Yat-sen University, Zhuhai, China

**Keywords:** cognitive function, finger maze test, visual stimulation, memory, attention, stress

## Abstract

Stress in life is ubiquitous and unavoidable. Prolonged exposure to severe stress can lead to physical intolerance and impair cognitive function. Non-human primates are considered to be the best animal model for studying cognitive function, especially memory and attention. The finger maze test, with the advantages of short training time and lower cost, is recommended to evaluate learning and memory in non-human primates. In this study, we modified the finger maze test method to evaluate the cognitive function of single-housed cynomolgus monkeys. The flexibility and attention of cynomolgus monkeys were assessed by performing the complex task test and the stranger intrusion interference test, respectively, which increased the difficulty of obtaining rewards, and the ability of long-term memory was also evaluated by the memory test. Furthermore, the changes in cognitive function of the cynomolgus monkeys were tested by using the finger maze test after audio-visual stimulation, and the changes in the cortisol levels during stimulation were also analyzed. We found that, after completing the learning test, there was no significant decrease in their success rate when monkeys processed multitasks at the same time. In the stranger intrusion interference test, all subjects were distracted, but the accuracy did not decrease. The monkeys completed the memory tests in the 1st and 2nd months after the learning tests, with a high success rate. However, the success rate decreased significantly at the end of the 4th month. During audio-visual stimulation, the plasma cortisol level significantly increased in the first 2 months and was maintained at a high level thereafter. One month after audio-visual stimulation, the accuracy of the memory test was significantly reduced, and the total time of distraction was significantly prolonged. In conclusion, chronic audio-visual stimulation can increase blood cortisol levels and impair cognitive function. The modified finger maze test can evaluate many aspects of cognitive function and assess the changes in the cognitive function of adult cynomolgus monkeys under stress.

## Introduction

Cognitive function is gaining increased attention since cognitive impairment can affect the routine life and social communication of an individual (Gorelick et al., 2016). Recent studies revealed that not only are aging (Fu et al., 2018; Zhang et al., 2019) and many diseases (Rogan and Lippa, 2002; Dodd et al., 2010; Komiyama et al., 2017; Prakash et al., 2017; Silverberg et al., 2020; Dobretsova and Derakshan, 2021; Hooke et al., 2021) associated with cognitive decline, but psychological stress is as well or impairment (Sun and Alkon, 2014; Reshetnikov et al., 2020).

Stressful situations tend to disable the cognitive capabilities of people, which may bring many adverse consequences, such as the inability to maintain healthy lifestyle choices (Schneiderman et al., 2005) or poor performance at important decisive moments (Yu, 2015). Learning, memory, and decision-making abilities, which are important cognitive capabilities, are weakened by stress (Schwabe and Oliver, 2013; Porcelli and Delgado, 2017; Nitschke et al., 2020). When the body challenges some additional stimuli, stress occurs (Chrousos, 2009), which can lead to an increase in cortisol (Russell and Lightman, 2019; Sabia and Hupbach, 2020; Smeets et al., 2021). Under the condition of prolonged exposure to stress, the high level of cortisol in the long-term can cause the loss of neurons, especially in the hippocampus (McEwen and Sapolsky, 1995), and the loss of CA3 pyramidal neurons of the hippocampus leads to significant morphological changes (Joëls et al., 2007; Merz et al., 2020). Experiments corroborated that the physical or psychological stress depression model can cause metabolic changes in the hippocampus (Kavushansky et al., 2009), and behavioral abnormalities and affective disorders post-stress are likely to be related to functional impairment of the hippocampus (Jacobs et al., 2003). Cortisol is frequently investigated as a biomarker of stress and a potential intermediary between stress and impaired brain function (Law and Clow, 2020). Immediately after reaching a peak of corticosteroid hormone levels, alertness and attention are increased, and brain areas including simple behavioral strategies and emotional responses show enhanced activities (Joëls, 2018). In addition to the slow response of glucocorticoids, stress events also affect cognitive function through the rapid response of catecholamines (McEwen and Sapolsky, 1995), which play a key role in alertness, directionality, selective attention, memory, and other reactions (Southwick et al., 1999). An animal experiment showed that variable stress impaired attentional performance in the sustained attention task (Eck et al., 2020). A study based on Eriksen's flanker task investigated the influence of psychological stress, indicating elevated vigilance levels under stress and more intensive attention control (Qi and Gao, 2020). However, the patients with stress-related exhaustion performed poorly in executive function and complex attention (Krabbe et al., 2017), and working memory and attention could not be recovered even after 3 years (Jonsdottir et al., 2017).

The evaluation of the cognitive function of non-human primates is of great importance for studying the process and mechanism of cognitive changes caused by diseases or drugs (Mishra et al., 2020; Sekioka et al., 2020), but the methods to assess cognitive function are very limited (Inoue et al., 2014). The Wisconsin General Test Apparatus (WGTA) is mainly applied to test the learning and memory ability of monkeys (Makori et al., 2013; Rose et al., 2015), and the Cambridge Neuropsychological Test Automated Battery (CANTAB) is recommended to evaluate reaction time and memory and executive functions (Spinelli et al., 2004; Wright and Taffe, 2014; Lacreuse et al., 2018; Marino and Levy, 2019), but the disadvantages are testing only one monkey at a time and requiring a long initial training period. The finger maze test was used to assess learning and memory in non-human primates (Tsuchida et al., 2003). In a recent study, the optimized finger maze test showed a few disadvantages in solving problems, including long training time, high cost, and incompatibility for use by several monkeys simultaneously, although it was confirmed that monkeys were able to learn new rules, remember them even for an extended period of time, and successfully retrieve them 2 months later (Kim et al., 2020). However, it is unclear how soon the monkeys will gradually forget the rules of the finger maze test. The finger maze test was used in experiments about toxicity and aging to assess the changes in learning and memory (Itoh et al., 2001; Inoue et al., 2014), but whether it can be applied to evaluate the changes in the cognitive function of adult cynomolgus monkeys under stress has not been studied.

We found that, when monkeys saw strangers, even during the period of eating, their attention would be attracted, and that, they continued eating after watching the activities of strangers for a short time. We speculated that, after chronic and intense stimulation, the alertness of the monkeys would be increased, and the time of attention diversion might be longer. Therefore, we designed a stranger intrusion interference test to determine and compare the changes in the time of distraction and the success rate of completing the finger maze test after stimulation. A previous study reported that visual stimulation (monkey picture) induced an increase in blood cortisol levels in cynomolgus monkeys through mild stress and enhanced their cognitive behavior (Woo et al., 2018). Because high-intensity and long-term stress are associated with cognitive decline (Sandi, 2013), and to expose monkeys to more intense psychological stimulation, we used the method of audio-visual stimulation (tiger video) and measured their plasma cortisol level. We assessed the effects on the cognitive function of adult cynomolgus monkeys, especially memory and attention, induced by audio-visual stimulation by using the finger maze test. In addition, we added the complex task test to measure the flexibility of monkeys and evaluated their memory for a longer period. In this study, we attempted to apply the finger maze test to evaluate the multiple aspects of cognitive function and observe the changes in cognitive function after stimulation.

## Materials and methods

### Subjects

All procedures involving animals and their care were approved by the Institutional Animal Care and Use Committee of Yuanxi Biotech Inc., Guangzhou, China (YXSW-2020-003). Four male cynomolgus monkeys (age: 6 years, weight: 7.3–9.7 kg) were selected for the present study. They were housed in individual primate cages in a climate-control room with the temperature maintained at about 24 ± 2°C and a 12-h light–dark cycle. Monkeys were fed commercial monkey chow and fresh water *ad libitum*, supplemented with fresh fruits.

### Hand dexterity test

Monkeys with defective finger flexibility may affect the training time and success rate of completing the finger maze test and thus may be unable to correctly evaluate cognitive function. Therefore, the detection of finger flexibility is needed to ensure that monkeys have no motor dysfunction. When an 8-well test board, with each well (1 × 1 × 1 cm) holding apples, was attached to the front of the cage, the monkeys quickly took out apples with their fingers and ate them (**Figure 3A**). The hand dexterity test (HDT) was conducted on the monkeys after three training sessions, and the time to remove all the apples was calculated by recorded video (JVC GZ-E765-T).

### Finger maze test

**Figure 1:**
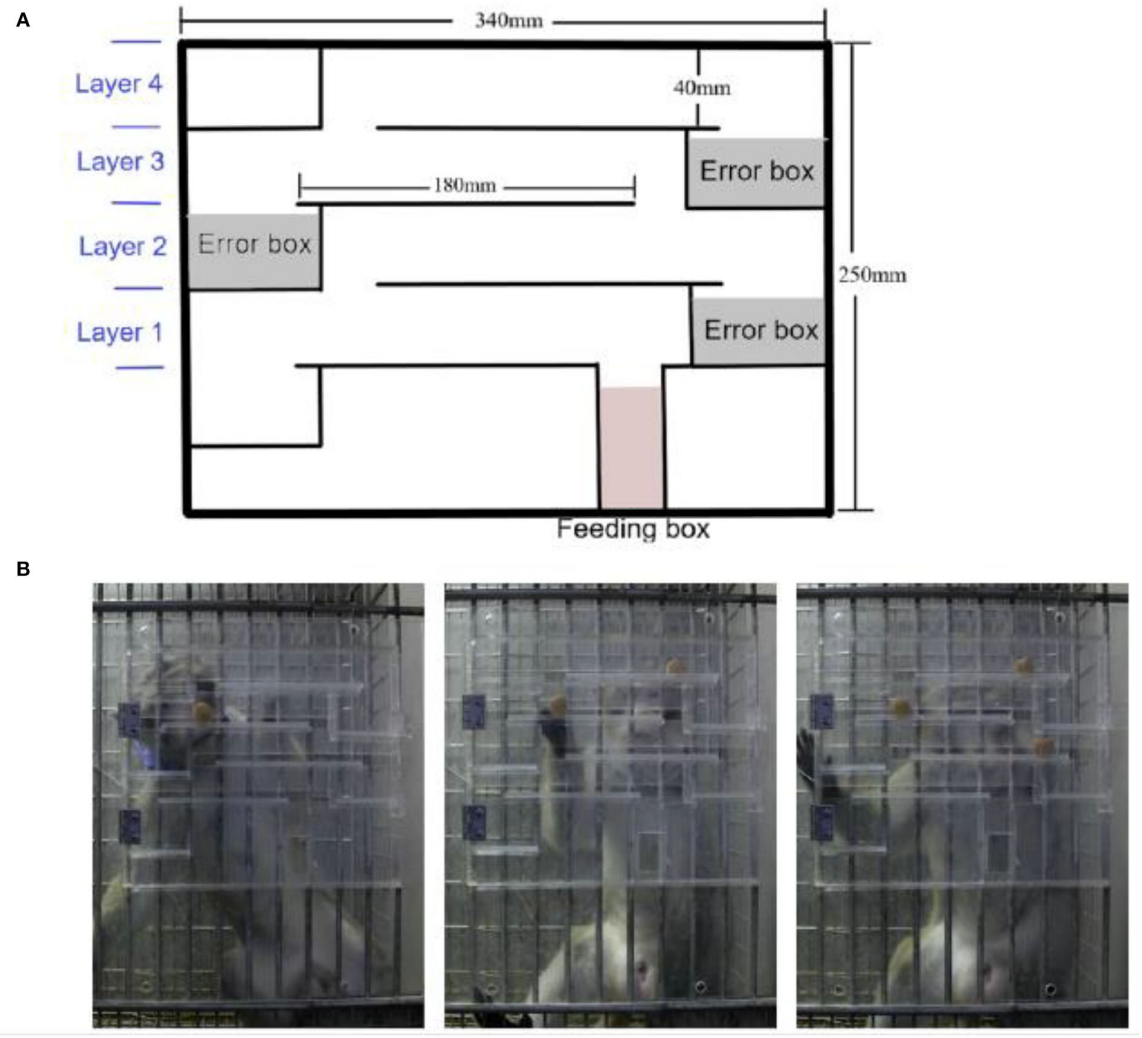
Application of the modified finger maze test. **(A)** Schematic diagram of the finger maze apparatus. **(B)** Different numbers of rewards were placed, one on each layer.

As previously reported (Kim et al., 2020), the finger maze device was modified to fit the structure of the monkey cage size in the lab and could be suspended in front of the cage. The finger maze device is made up of four layers, each with an error box and an additional feeding box at the bottom. The transparent acrylic panel in the front of the device could be opened to place rewards at any position (Figure 1A). The monkey moved the reward with his fingers, and then the reward entered the lower layer and finally dropped into the feeding box when the monkey chose the right direction, which otherwise fell into the error box, which was inaccessible. During the test, the experimenter stood in front of the cage where the device was suspended, and after the monkey got the reward and ate it, another reward was placed. The reward in the experiment was commercial monkey chow.

### Training

**Figure 2:**
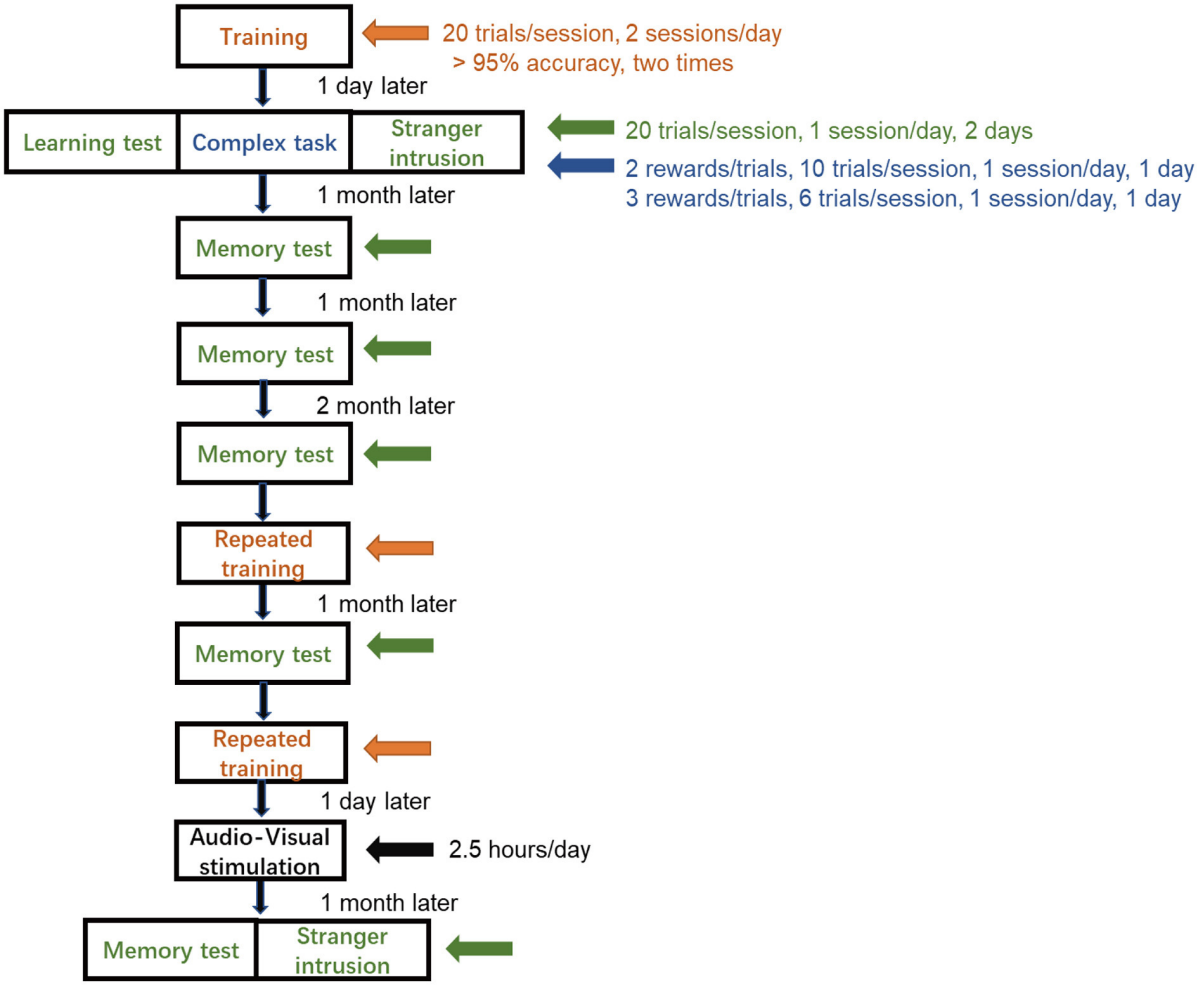
Schedule of finger maze test. The monkeys did not complete the training until the success rate achieved more than 95% accuracy for two consecutive sessions and proceeded to the learning test, the complex task test, and the stranger intrusion interference test, each for 2 days. Memory tests were performed at a specific time. Repeated training was followed by audio-visual stimulation, and then, after 1 month, the memory test and the stranger intrusion interference test after stimulation were carried out.

To acclimate the monkeys to the finger maze device, it was hung in front of the monkey cage for 2 days before the training. A training session was conducted two times a day, and the session consisted of 20 trials (Figure 2). When the reward was on the first layer, the monkey moved it to the channel connecting the feeding box on the left with the right finger, and then the monkey got the reward. The criteria for success in each layer was to achieve 95% accuracy and was completed two times by this standard. When the monkey passed the first layer, it entered the second layer of training, which required the monkey to move the reward in the opposite direction and through the channel connecting to the first layer, and then do the same way in the first layer. Monkeys completed the third and fourth layers of training in the same way. The training trials of completing each layer were recorded. When the monkeys met the criterion of the fourth layer, they underwent other tests.

### Learning test

A learning test determined whether the monkeys had mastered the rules and succeeded in the finger maze test (Figure 2). The subjects were tested for 2 days, with 20 trials a day. A reward was placed in a pseudo-random position, and the monkey needed to determine the location of the reward and move it in the right direction to the feeding box. The number of rewards a monkey obtained was the number of successes. The success rate was defined as the success number divided by the trial number.

### Complex task test

Multiple rewards were placed in pseudo-random positions on different layers at the same time, and only one was placed on each layer (Figure 1B). Monkeys identified the location of multiple rewards at the same time, judged the sequence and direction of moving the rewards, and finally obtained multiple rewards. Two rewards were placed in the device simultaneously and one session containing 10 trials with 20 rewards was performed on the first day. The other session with three rewards positioned at the same instant with 6 trials and 18 rewards, was conducted on the following day (Figure 2). The success rate was calculated in the same way.

### Stranger intrusion interference test

When another experimenter unfamiliar to the monkeys came into sight of the monkeys, the stranger intrusion interference test began, and then the former experimenter started to place rewards in the device (Figure 2). The stranger walked back and forth at a speed of 1 m/s within the sight of the monkey at a distance of 3 m from the cage and stared at the monkey for 1 s when he came to the front of the monkey cage, looking in the direction of his walk at other times (**Figure 5A**). The attention of the monkey was attracted by the activities of the stranger, and it stopped testing for a few seconds. Rewards were placed in the same way as the learning test. After enough tests, when each monkey performed the finger maze test attentively, the subject did not make the reward stay in a fixed position for more than 1 s. A distraction was defined as the hand of the monkey remaining stationary for more than 1 s without moving toward the reward. The time of distraction was the time it took for the reward to stop moving until it started moving again and was longer than 1 s. The behaviors of each subject were recorded by a video camera. Two experimenters, who had not seen the subjects before, calculated the time of distraction by applying Beecut software. The success rate and the total time of distraction in one session were measured.

### Memory test

Memory tests were carried out at five time points in the same way as the learning test: three tests in the first, second, and fourth months; one was conducted 1 month after the repeated training and the other was done after audio-visual stimulation (Figure 2). The success rate was also measured as a learning test protocol.

### Repeated training

To increase the success rate and to achieve a consistent level, all monkeys underwent repeated training (Figure 2). Each monkey was trained twice a day with 20 trials per session, while the reward was placed in a pseudo-random position, and eventually, the success rate reached more than 95% accuracy for two consecutive sessions.

### Audio-visual stimulation

To exert chronic stress on monkeys, the approach of audio-visual stimulation was conducted for 1 month (Figure 2). The four monkeys were trained to sit in monkey chairs before being subjected to audio-visual stimulation. The subject sat 0.6 m away from the liquid crystal display (47 × 26 × 40 mm, PHILIPS, model 226V4L), which could not be touched, and watched a blank screen for 30 min, and then the edited video of tiger activities, accompanied by tiger roars (top sound of 80 dB) for 2 h each day, was displayed. High-definition videos of tigers were downloaded from the Internet, and clips including direct-gaze (signaling threat), roaring, showing fangs, and chasing or biting animals were combined into a 40-min video. Each video was played in full-screen mode, and one trial was composed of a 40-min video. After the first trial, there was no interval and it was followed by the second trial; a total of three trials was conducted (**Figure 8A**). The edited video was changed every half a month because watching the same video might attenuate the effect of audio-visual stimulation.

### Cortisol collection

Because of the circadian rhythm of cortisol levels (Mohd Azmi et al., 2021), we collected blood samples from the cubital vein of cynomolgus monkeys at 10:30–10:40 am during the study period from 6 March to 5 April. The blood sample was drawn into a blood collection vessel containing heparin, mixed, and then centrifuged (3000r/min,15 min), and the plasma was separated and stored in a refrigerator at −80 °C until analysis. We collected blood samples the day before stimulation and every week during audio-visual stimulation. The cortisol levels in plasma were determined by enzyme-linked immunosorbent assay (ELISA) using standard commercial kits (Jiangsu Meimian Industrial Co., Ltd., Jiangsu, China).

### Statistical analysis

A one-way ANOVA was used for multiple comparisons followed by an LDS *post hoc* test for each comparison in the learning and memory tests and the cortisol levels. The results of the learning, complex task, stranger intrusion interference tests, and data after repeated training were analyzed by paired sample *t*-test. The total time of distraction was analyzed by the Kruskal-Wallis test. All statistical analyses were performed by using SPSS V18 for Windows statistical package.

## Result

### Hand dexterity test

**Figure 3:**
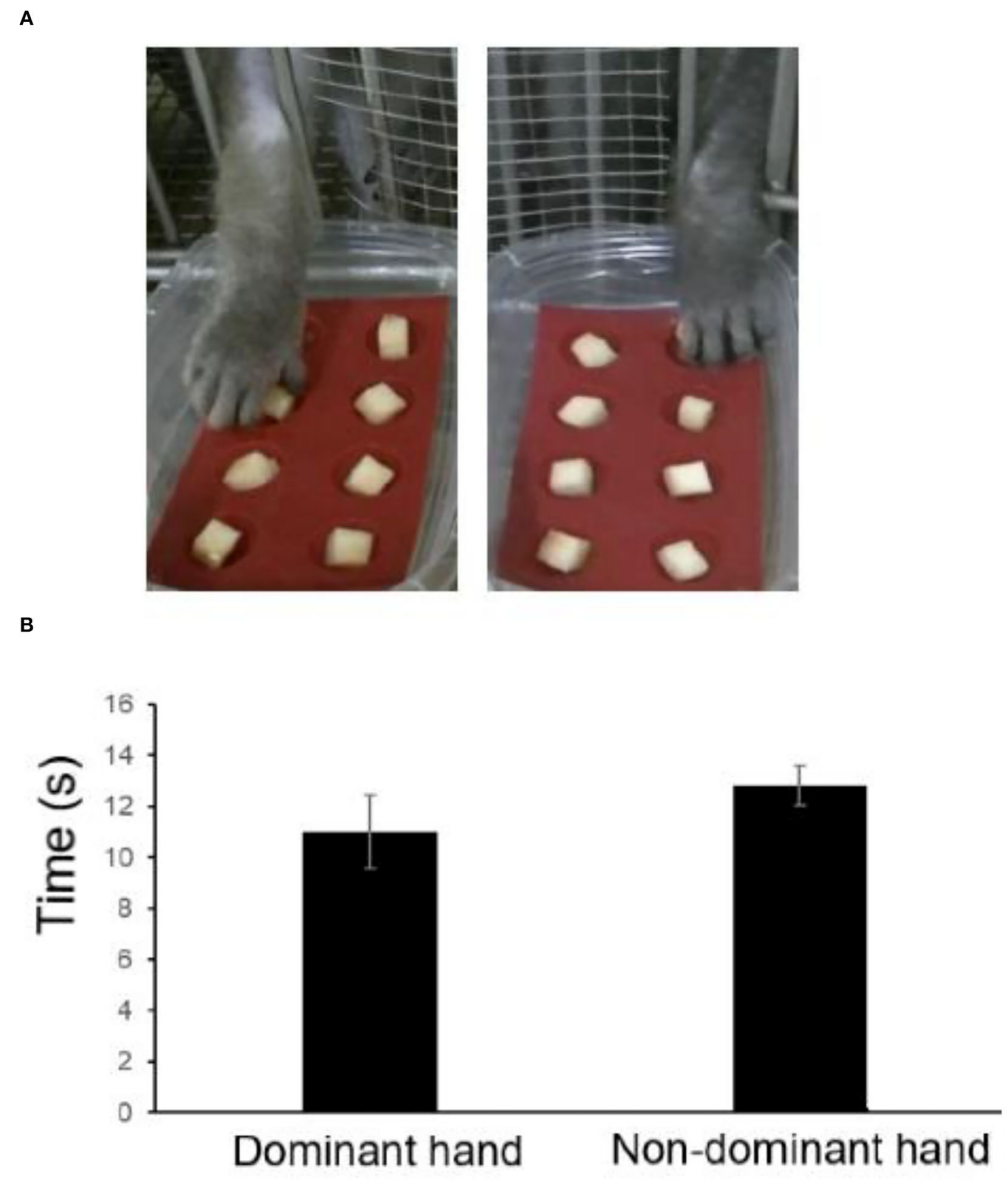
Retrieval time of each hand using the hand dexterity test (HDT). **(A)** Representative images. **(B)** Retrieval time estimated by the HDT.

It took a shorter time to retrieve rewards for the dominant hand, while the non-dominant hand took longer. The monkeys could quickly get all the apples with both hands (8.6–13.75 s), which showed that the finger movements of the monkeys were not impaired, thus we did not consider the difference between each monkey (Figure 3).

### Training

**Figure 4:**
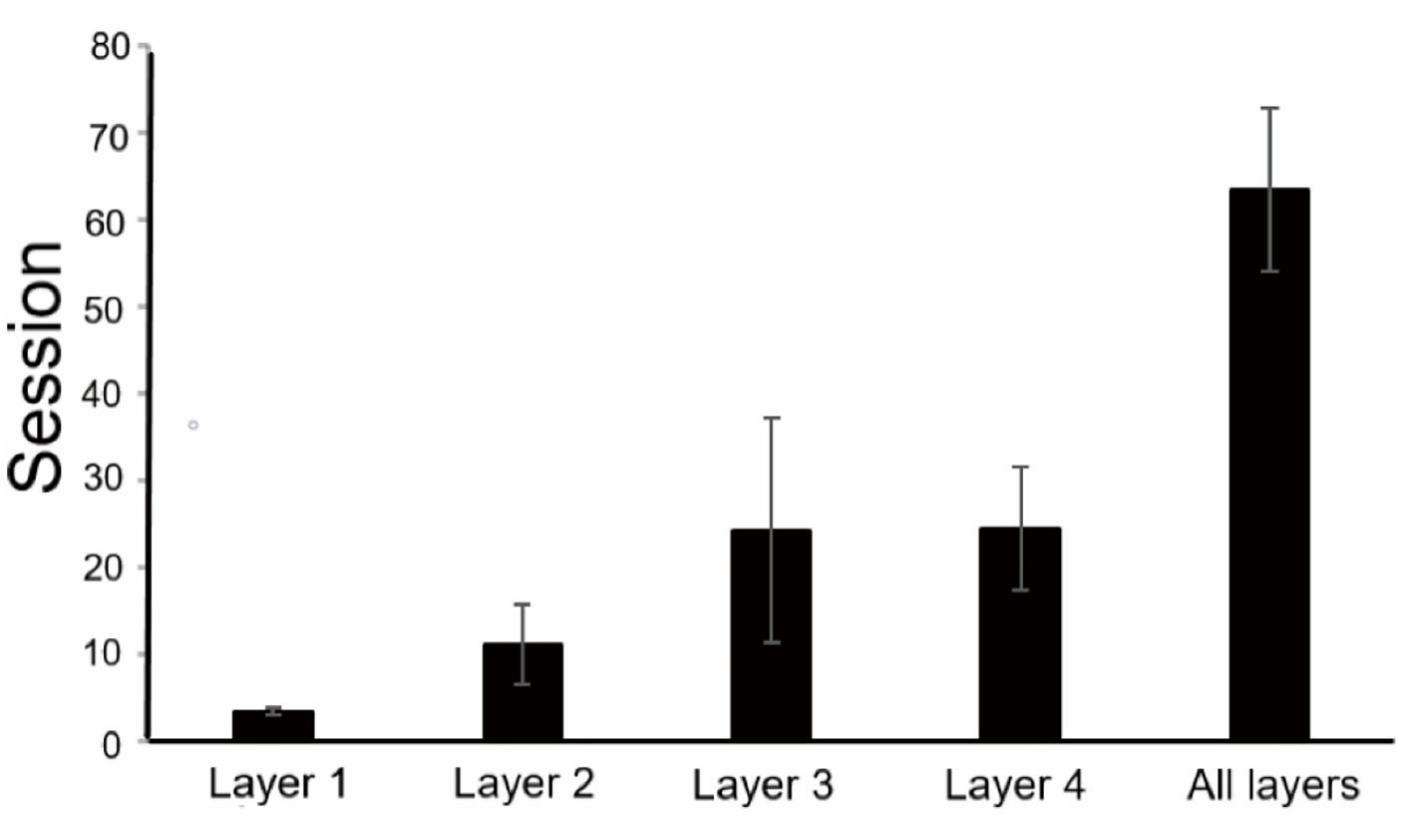
Training results of the finger maze test. An average number of sessions to reach the criterion for each layer and the total number of sessions to complete training.

The time taken to reach the success standard of each layer was different (Figure 4), and the first layer was the shortest, with an average of 3.5 sessions, and the third and fourth layers were longer, with an average of 24.25 and 24.5 sessions, respectively. As the number of layers was added, the difficulty as well as the time it took to reach the success standard increased. Total training took an average of 63.5 sessions.

### Learning test, complex task test, and stranger intrusion interference test

**Figure 5:**
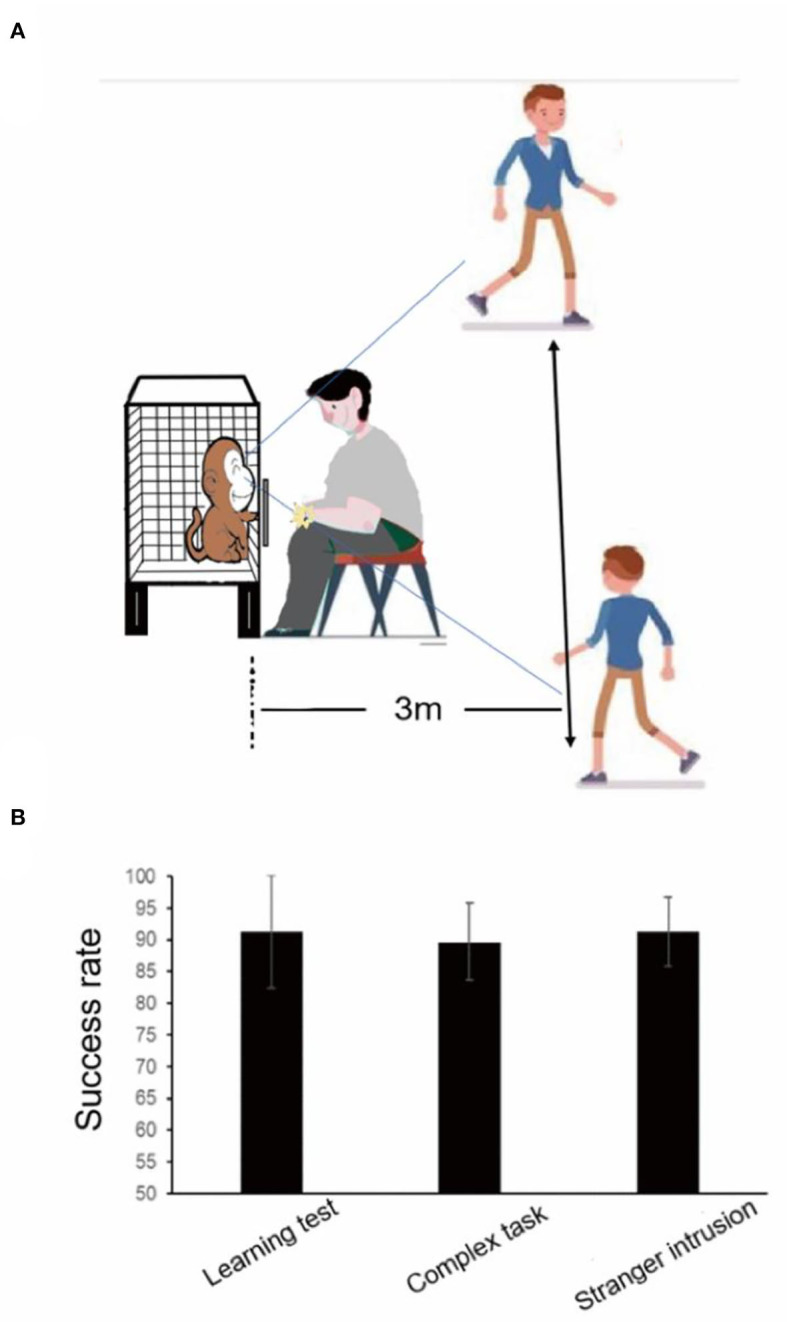
Results of the learning test, the complex task test, and the stranger intrusion interference test before audio-visual stimulation. **(A)** The stranger intrusion interference test paradigm. **(B)** There was no statistically significant difference between the learning test and the complex task test and the stranger intrusion interference test.

The learning test was performed two times, and the average success rate of the learning test was 91.25%, indicating that the monkeys were adept at using the rules of the finger maze and completed the finger maze test successfully. The complex task test was conducted the next day after the learning test, and two or three rewards were placed in the apparatus at the same time. In a total of 10 trials in one session, two rewards were placed at the same time on the first day when they were carried out, and for a total of 18 rewards, 6 trials were performed on the following day. The average success rate was 89.65%. Although the accuracy decreased, there was no statistically significant difference between the test results of the learning and complex task tests (*p* = 0.545, paired *t*-test), suggesting that after learning the rules of the finger maze, the monkeys were able to flexibly apply them to the test of obtaining multiple rewards at the same time (Figure 5B). When monkeys performed the complex tasks in the first trial or the second trial, they would prefer to move the top layer reward. The upper reward moved closely to a lower one until it was blocked, and then the monkey chose to move the reward on the lower layer which could be easily obtained. In the following trials, they would master this rule and move the reward on the lower layer first.

When the stranger came into sight of the monkey, especially when looking at it, the subject no longer focused on completing the test, stopped poking the reward with their fingers, and then continued to move the reward after observing the activities of the stranger for a few seconds. In the stranger intrusion interference test, the attention of the monkey would shift to the moving stranger many times, and it was unable to continuously concentrate on completing the test. However, the average success rate was 91.25%, and the success rate was not decreased between the learning test and the stranger intrusion interference test (Figure 5B, *p* = 1.0, paired *t*-test).

### Memory tests

**Figure 6:**
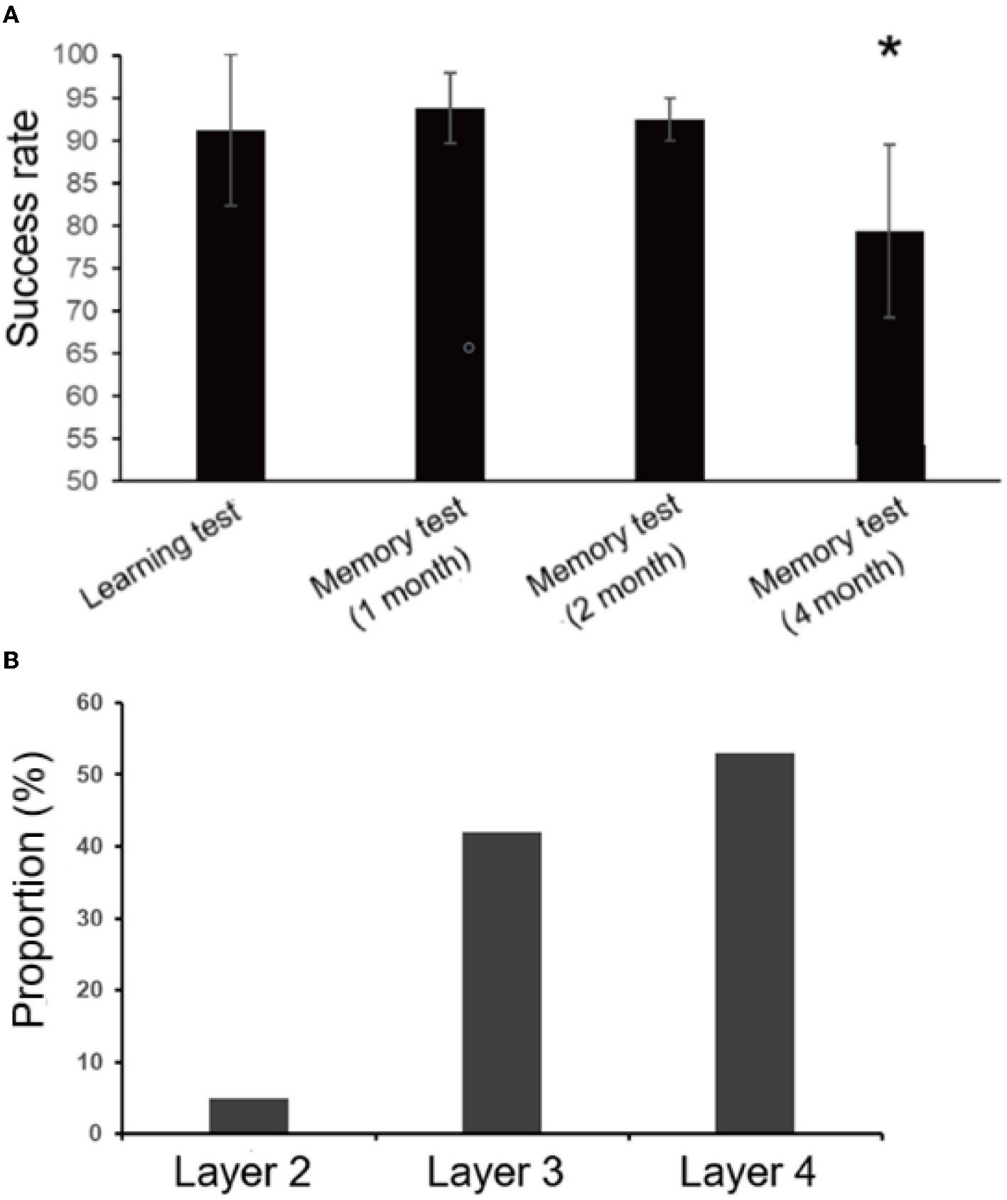
Results of the learning and memory test. **(A)**There was no significant difference between the learning test and the memory test in the first 2 months, but the success rate decreased significantly (^*^p < 0.05) in the fourth month. **(B)** Most of the rewards that fell into the error box were placed on the fourth floor (53%), and the least on the second floor (5%) in the the memory tests. Rewards placed on the third layer account for 42%.

At different times after the learning test, monkeys were tested for memory. After 1 and 2 months, the average success rates of memory tests were 93.75 and 92.5%, respectively. The average success rate of the memory test in the first 2 months was higher than that of the learning test (91.25%), which may be because the complex task test and the stranger intrusion interference test enhanced the monkeys' memories of the rules in the finger maze test. These results showed that, even after 2 months, the monkeys were able to complete the finger maze test with high accuracy, and they remembered the rules of the finger maze test. However, the success rate decreased significantly in the fourth month compared with other times (one-way ANOVA, *F*_3, 31_ = 6.0, *p* = 0.003, followed by LSD *post hoc* test, *p* < 0.05). The average success rate was 79.38% (Figure 6A), indicating that the monkeys gradually and partially forgot the way to get rewards. Of the rewards dropped into the error box, the rewards placed on the fourth layer were the most (53%), followed by the third layer (42%), and the second layer was the least (5%) in the memory tests (Figure 6B). The uncertainty about the direction where the monkeys would move the rewards increased the error rate.

### Repeated training and subsequent memory test

We speculated that, when monkeys could complete the finger maze experiment with high accuracy, the success rate might decline more significantly after a period of time and perhaps more obvious changes could be detected in the following stress experiment. To observe the changes in long-term memory more accurately, repeated training was performed to make the success rate reach a high level (≥95%). Each monkey took a different time to reach the standard (5, 6, 8, and 12 sessions, respectively).

**Figure 7:**
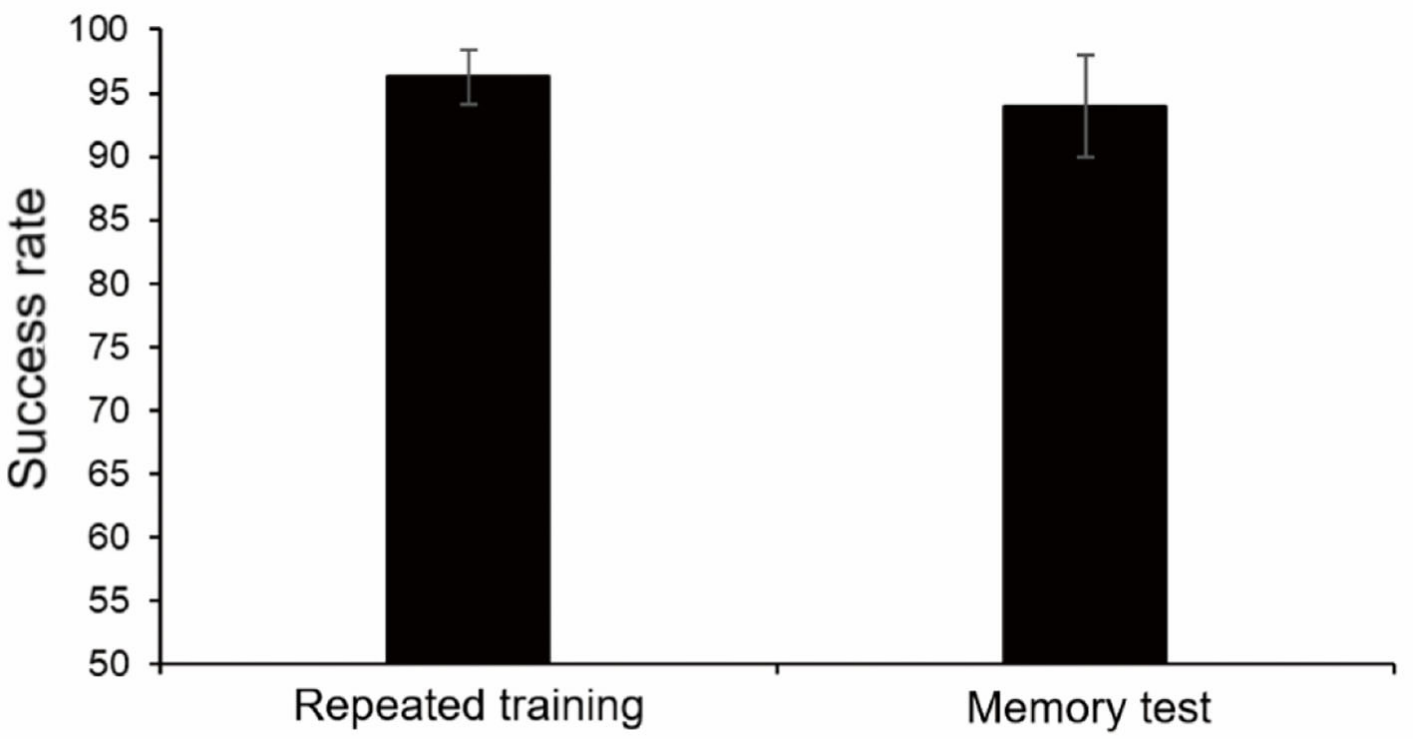
Results of repeated training and memory test 1 month later. There was no statistically significant difference.

After 1 month, the memory test was carried out, and the success rate decreased. However, there was no statistically significant difference between repeated training (96.25%, average) and memory test (94.0%, average, *p* = 0.17, paired *t*-test). It showed that repeated training had consolidated long-term memory and increased proficiency, and monkeys could also maintain a high success rate after a month (Figure 7).

Before the audio-visual stimulation, we repeated the training. After two to four sessions of training, the success rate of completing the finger maze test exceeded 95% (97.5%, average). This indicated that the success rate of monkeys could be maintained at a high level for a long time and rapidly improved.

### Tests after audio-visual stimulation

**Figure 8:**
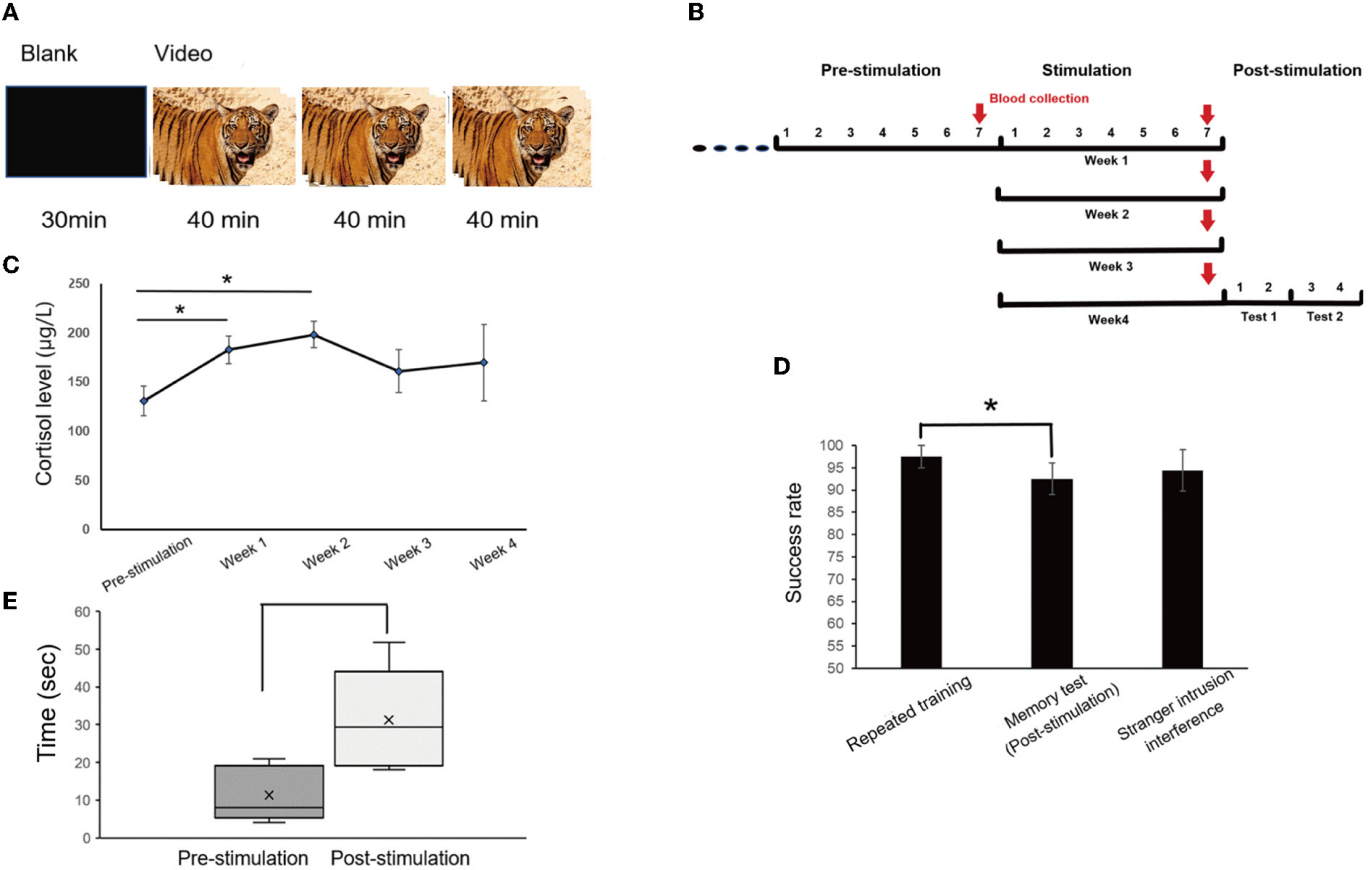
Method of visual stimulation and the results after visual stimulation. **(A)** The daily session was for 2.5 h per day, with blank images for 30 min and the video for a total of 2 h, including three trials, each for 40 min. **(B)** Timeline of audio-visual stimulation and blood collection. Blood samples were taken once, the day before stimulation and every 6 days during stimulation, for a total of five times. **(C)** Cortisol analysis. Cortisol levels increased significantly at the end of the first and second weeks (one-way ANOVA, *F*_4, 19_ = 3.731, *p* = 0.027, followed by LSD *post ho*c test, ^*^p < 0.05). **(D)** There was a significant difference between repeated training and memory tests after stimulation (^*^p < 0.05). **(E)** The distraction time was significantly prolonged after audio-visual stimulation in the stranger intrusion interference test (^*^p < 0.05).

The subjects were used to sitting in the monkey chairs before stimulation. A previous study reported that, compared with the week before stimulation, the cortisol level of monkeys watching the monkey pictures was significantly increased, indicating that the monkeys had a mild stress response. However, human and animated pictures did not increase the cortisol level of monkeys, although there was the same trend (Woo et al., 2018). To make monkeys exert chronic stress, videos of tigers were selected. The contents of audio-visual stimulation were blank images without sound for 30 min and videos of multiple clips of tigers with roars for a total of 2 h, and a 40-min video was played 3 consecutive times (Figure 8A). Blood samples were collected from monkeys at the end of the pre-stimulation week and after the end of each stimulation week (Figure 8B). During the audio-visual stimulation, the plasma cortisol level of the subjects increased significantly compared with physiological conditions (one-way ANOVA, *F*_4, 19_ = 3.731, *P* = 0.027, followed by LSD *post hoc* test, *p* < 0.05). The cortisol levels statistically increased significantly during the first 2 weeks, peaked in the second week (*P* < 0.05), and then decreased slightly in the third and fourth weeks, but they were still at a high level (Figure 8C). The cortisol levels did not show any statistical significance in the last 2 weeks, which may be due to a small number of samples but still represented a state of stress.

After 1 month of continuous audio-visual stimulation, memory tests were carried out one session a day for 2 days, and on the third day, the stranger intrusion interference test began in the same way. One test was conducted every day for two consecutive days, for a total of four days. There was a significant difference between repeated training (97.5%, average) and the memory test (92.5%, *P* < 0.05, paired *t*-test), but no statistically significant difference between repeated training and the stranger intrusion interference test (94.38%, *p* = 0.402, paired *t*-test, Figure 8D). Moreover, compared with the normal physiological state (pre-stimulation), the time of distraction under stress state (post-stimulation) was significantly prolonged in the stranger intrusion interference test (*p* < 0.05, Kruskal-Wallis test, Figure 8E), suggesting that monkeys were more alert to the threat of active intruders and spent more time to transfer attention to strangers.

## Discussion

In previous reports, rhesus monkeys took a shorter time to complete the training, whose success criterion for each step was completing 8 consecutive trials without failure (Kim et al., 2020). In our experiment, monkeys were trained for more trials in the finger maze tests, which might be related to the species of monkeys and success criteria. Forgetting is a physiological phenomenon in which monkeys fail to retrieve valid information to obtain rewards as time passes (Reynoso-Cruz et al., 2021). To distinguish it from amnesia after stress, we evaluated the long-term memory of four untreated cynomolgus monkeys, and they all were able to successfully apply the rules for 2 months, which later was consistent with the results of a previous study (Kim et al., 2020). Timely review and necessary practice can reduce the possibility of forgetting and improve memory ability (Kornmeier et al., 2022). In our findings, the success rate of the memory test in the first 2 months was slightly higher than that of the learning test. This may be because the complex task test and the stranger intrusion interference test after the learning test play the role of practice, promoting information storage (the memory of the finger maze rules) and the consolidation of memory in the brain. However, the success rate decreased in the fourth month after the learning test, suggesting that the memory of the rules could not be retained for 4 months, but we could better measure and compare the memory changes of animal models within 2 months.

After completing the learning test, the monkeys successfully completed the complex task test, which increased the difficulty of the finger maze test, suggesting that monkeys had rapid reflexes and proficient and flexible use of finger maze rules. The unfamiliar human intrusion was considered a mild stress stimulus (Peterson et al., 2017), which was designed to measure the response of the monkey to a potentially threatening social stimulus and was used to assess anxiety (Coleman and Pierre, 2014). The monkeys reacted to intruders entering the room and approaching the monkey cage with threatening behavior, fear responses, and/or freezing (Coleman and Pierre, 2014). During the experiment, we found that, once the reward was presented, the monkeys would concentrate their attention on completing the finger maze test until they got food. When the intruder appeared, the monkey stopped moving the reward and just stared at the stranger for a short time and then continued to complete the test. This might be because the combined effects of the threat of strangers and the temptation of food changed the original behavior of monkeys, making the attention of the monkeys turn to an intruder for a short time. The present study suggested that mild stress tended to facilitate cognitive function without impairing accuracy or interference control (Shields et al., 2019), particularly in implicit memory or simple tasks (Sandi, 2013), which was illustrated by the fact that, in our experiment, the success rate of completing the finger maze test on the stranger intrusion interference test did not decrease. Perhaps strong threats will affect the accuracy of the monkeys in completing the finger maze test, such as the aggressive actions of strangers.

Psychosocial stress is associated with the impairment of broad cognitive functions and long-term abnormalities in spatial working memory and attention (Olver et al., 2015). Long-term exposure to stress hormones will affect the brain structure related to cognitive and mental health. However, the specific effects on the brain, behavior, and cognition depend on the duration of exposure (Lupien et al., 2009). Glucocorticoids are thought to affect hippocampal-dependent spatial memory and dorsal striatal-dependent programmed memory (Siller-Pérez et al., 2017). Stress can impair the hippocampal-dependent system and then the striatum controls behavior (Schwabe and Wolf, 2012). The increase in the cortisol levels induced by psychological stress can damage spatial memory and procedural memory. However, only the increase in cortisol can cause poor performance in declarative memory and spatial thinking tasks, but there is no obvious abnormality in procedural memory tasks (Kirschbaum et al., 1996). In our experiment, the cortisol levels were significantly increased and were maintained at a high level during audio-visual stimulation, indicating that the monkeys were in a state of stress. The previous report showed that visual stimulation (monkey, human, or animation pictures) caused mild stress by a slight increase of about 24–31% in blood cortisol levels (Woo et al., 2018). Our results indicated that audio-visual stimulation (tiger video) caused more severe stress of about 23.3–51.8% in blood cortisol levels than viewing pictures. After 1 month of continuous stimulation, the memory of the subjects decreased significantly, which may be related to the damage of the hippocampus and the striatum caused by sustained high levels of stress hormones.

The relationship between stress and attention is far from fully understood. Some studies reported better attentional choices during and after stress, some reported poor attentional choices, and others reported that stress had no effect on attentional choices at all (Larra et al., 2016). This may be related to the intensity and duration of stress, and high-intensity and long-term stress could damage cognitive function (Sandi, 2013). Stress could do great damage to intention-based attention allocation, resulting in strong distraction in the process of information selection of attention (Sänger et al., 2014). In addition to the reason that stress affects attention (Eck et al., 2020), high alertness may be another reason why the monkeys are distracted. Patients with traumatic stress disorder often have symptoms such as hypervigilance and exaggerated startle (Southwick et al., 1999). The alertness of the monkeys might be increased by continuous audio-visual stimulation, coupled with the impact of stress on attention, which ultimately leads to the extension of the time to transfer attention to strangers. However, the accuracy of the stranger intrusion interference test did not decrease significantly immediately after the memory test, because the memory test after stimulation could be thought of as a process of memory retrieval. Memory retrieval triggers a series of processes that reinforce stored information and activate the consolidation of a second memory, which can stabilize the expression of the original memory (Suzuki et al., 2004). Thus, the monkeys performed better in the stranger intrusion interference test than in the memory test after stimulation.

Although the number of subjects was relatively small and may affect the power of statistical tests, most of the current results were consistent with the previous studies. Non-human primate attention was usually measured by eye movements (Recanzone and Wurtz, 2000; Goldberg et al., 2006), but it was not suitable for free-moving monkeys. We used a very simple method to assess attention, which was a rough estimate, but it could indirectly represent the time of distraction.

In conclusion, audio-visual stimulation can induce stress in non-human primates and impair cognitive function. The finger maze test is an effective tool to evaluate the cognitive function of cynomolgus monkeys, which could be used to measure the changes in cognitive function due to stress in non-human primates, and an attempt could be made to use this tool in other animal models.

## Data availability statement

The original contributions presented in the study are included in the article/supplementary material, further inquiries can be directed to the corresponding author/s.

## Ethics statement

The animal study was reviewed and approved by Institutional Animal Care and Use Committee of Yuanxi Biotech Inc.

## Author contributions

SW and YH designed experiments. YH, HW, and YD carried out experiments. HJ analyzed experimental results. YH, CY, and YL wrote the manuscript. All authors contributed to the article and approved the submitted version.

## Funding

This work was supported by Military Logistics Open Research Project (BWS19J012).

## Conflict of interest

The authors declare that the research was conducted in the absence of any commercial or financial relationships that could be construed as a potential conflict of interest.

## Publisher's note

All claims expressed in this article are solely those of the authors and do not necessarily represent those of their affiliated organizations, or those of the publisher, the editors and the reviewers. Any product that may be evaluated in this article, or claim that may be made by its manufacturer, is not guaranteed or endorsed by the publisher.
